# Transactions of the Provincial Medical and Surgical Association. (Statistical and Topographical Papers.)

**Published:** 1838-07

**Authors:** 


### BRITISH AND FOREIGN
### MEDICAL REVIEW,

FOR JULY, 1838.

### PART FIRST.

### Analytical and Critical Reviews.

### ART. I.

*Transactions of the Provincial Medical and Surgical Association.—London and Worcester.* Vols. I.—V. 1833-37. 8vo. (STATISTICAL AND TOPOGRAPHICAL PAPERS.)

IN the general notices of these volumes which we gave in former Numbers, we expressed our intention of referring to the statistical and topographical papers contained in them on some future occasion. This we now propose to do, with the view of bringing the important subjects to which they relate more prominently before our readers. It is not, however, intended to discuss the respective merits of these papers, on some of which, for obvious reasons, it is not for ourselves to pass any opinion. We are merely desirous of laying before our readers the more important facts which they contain, in the hope of impressing upon the profession the benefits likely to result from the cultivation of these branches of medical science, when conducted upon correct principles.

It has been reserved for the present age to estimate aright the value of statistical investigations; and it is now for the first time that we are fully alive to the necessity of reducing every branch of knowledge, susceptible of them, to the precision and rigorous exactness which numerical methods alone can give. No science, however rich in facts, can be said to have made real progress, until it is more or less brought within the sphere of mathematical calculation; and it is only in proportion as the rules and precision of what are termed the exact sciences are brought to bear upon it that any branch of knowledge can be said to approach perfection. This is a fact so evident in mechanical philosophy, that it need not here be insisted on; but that the natural sciences—those which are thought to depend more especially upon the accumulation of observations alone,—should be subject to the same rules, is perhaps not so clear to those who, from whatever cause, have not had the opportunity of tracing the recent progress of these sciences. The important aid, however, derived from the application of the doctrines of numbers and proportion to some of these must be familiar to all. It cannot have escaped the observation of any well-informed member of our own profession, that the precision introduced into chemistry, for instance, since the development of the atomic theory, has been attended with the most important and unlooked-for results; results quite independent of the hypothetical part of the theory, and arising solely from the application of the most simple elements of number and proportion to the facts of the science primarily deduced from experiment and observation. Errors of analysis have hence been detected, the existence of new principles has been rendered probable, and, by researches consequent thereon, demonstrated; while the whole science has assumed a precision and exactitude, an order and regularity, in strict accordance with that principle of harmony which pervades all creation. The same remarks apply to the natural sciences, more strictly so called. Astronomy, in particular, (which by some has been termed the Natural History of the Celestial Bodies,) is brought almost entirely within the sphere of physical science, by the application of the higher branches of mathematics to the investigation of the habitudes and relations of these bodies with respect to each other. Geology also seems now about to reap the advantages likely to result from the application of the same principles to the bewildering mass of facts and speculations, too often crude and sometimes wild in the extreme, with which the science is encumbered. The application of the principles of mathematical science in the development of the forms and structure of crystalline bodies by Hauy, and the doctrines of isomorphism lately advanced by Professor Mitscherlich, are of the first importance in the study of the mineral productions of our globe; while the researches into physical geography, by Humboldt and others, and recently in our country by Mr. Watson, show the importance of numerical methods of investigation, as applied to the vegetable and animal kingdoms. The statistics of Botany and Zoology, if they do not promise to make us better acquainted with the principle of life itself, will at least tend to develop some of those laws by which this principle acts, and through which it influences the organic world.

In a recent Number, we entered at some length into the consideration of the Numerical Method, as applicable to medical science; and, if we found reason to doubt the extent of this, as contended for by M. Louis and others, in regard to practical medicine, we entirely accord with that distinguished physician as to its immense value in every department of medical statistics. It is upon this method, indeed, that all medical enquiries of a statistical kind are mainly founded; and it is by the classification of the results obtained under its use, and the due comparison of these as they occur in different localities, that we expect to derive much knowledge as to the causes, the prevention, and the treatment of disease. When, therefore, we claim for statistical and topographical enquiries the earnest consideration of the medical profession, it is because we are convinced that these enquiries, if properly conducted, will throw light upon much that is now obscure, and reduce to precision much that is now doubtful in medical science. They will simplify our views as to the etiology of disease, by removing those elements from the enquiry into any particular case or class of cases which are foreign to it; and, in proportion as the causes are thus eliminated, the treatment adopted must, in like manner, become more simple, better adapted to its object, and consequently more efficacious.

From the nature of the materials in our possession, we must, on the present occasion, confine our attention chiefly to the southern and western parts of England, and especially to the western districts of Cornwall and the midland district of the Vale of Severn. In comparing these two localities together, we shall, for the most part, make use of the information contained in the volumes before us,—viz. of Dr. Forbes's Essay on the Land's End, for Cornwall; and of the valuable papers by Mr. Addison, of Malvern, Drs. Carrick and Symonds, of Bristol, Mr. Watson, of Stourport, Dr. Streeten, of Worcester, and Dr. Black, of Bolton. In addition to these, we shall avail ourselves of the facts contained in the Illustrations of the Natural History of Worcestershire, by Dr. Hastings; the Reports of the Worcester Infirmary and Dispensary, in the Midland Medical Reporter; and some other sources to which we shall allude as occasion requires.

The hundred of Penwith, or the district of the Land's End, is the western extremity of the county of Cornwall, and forms a projecting tract of a rude triangular shape, of which the sides washed by the sea are respectively about fifteen and twenty-five miles. The mean length of the district is twenty miles; the mean breadth about seven. From this it is evident that, in respect of climate, it must assimilate to that of a small island; as, with the exception of its eastern portion, or what may be termed its base, it is entirely surrounded by the waters of the Atlantic. The surface of this district is hilly, its shores precipitous; the mean height above the level of the sea probably between 400 and 500 feet; its highest hills not exceeding 1000 feet, although the hills beyond the boundary to the east are considerably more elevated. The geological formation is granite and slate of the primitive class; the alluvial deposits abundant in the valleys and low grounds, and partaking of the characters of the neighbouring rocks. There is a profusion of streamlets originating in the hilly tracts, but no river of any importance; the springs are copious and numerous, the water very pure. Both the granite and slaty rocks abound in metalliferous veins; and the extensive copper and tin mines, from the occupation which they afford to a large class of the inhabitants, form an important consideration in relation to medical statistics, as well as to other branches of statistical research. The climate of the Land's End is characterized by its equable temperature and its extreme humidity. The annual mean temperature at Penzance is 51°8; the greatest range, 65°; the mean annual range, 49°; the mean daily range, 6°7. The mean barometrical pressure is 29.61; the mean annual range, 1.68. Snow is seldom observed to fall, and in the vicinity of Penzance never remains on the soil for any length of time. The most prevalent winds are between north and west; those next in frequency between south and west. Thunder-storms are of rare occurrence. The productions of the soil also show the mildness of the climate; as many shrubs and plants grow in the open air, which, in most other parts of the kingdom, are killed by the cold and frosts of winter or early spring. The coolness of the summers, with the want of sunshine, however, prevents the due ripening of many of the late fruits,—as apricots, plums, &c.; and the harvests are by no means so early as in the midland counties.

The inhabitants are variously employed in agriculture, mining, handicraft and trade, and fishing. Of these the mining class is by far the most numerous, amounting to nearly one-half of the whole. They are exposed, in the mines, not only to severe work, frequently continued for a length of time, with a very scanty supply of light, and breathing a confined atmosphere, loaded with dust and impurities of different descriptions, but also to great humidity, conjoined with a considerable elevation of temperature, which increases with the depth of the mine and consequent atmospheric pressure. With respect to their domestic economy, many of the habitations of the poorer classes are built of clay and straw intermixed; but the cottages so constructed are generally warm and dry, however wretched in their external appearance. Fuel is scarce; the dress not peculiar; the diet composed chiefly of fish, pork, potatoes, and barley-bread. They were formerly much addicted to the use of ardent spirits, but there is now (since the introduction and spread of Methodism) a considerable improvement in this respect. Other features of their physical and moral condition we shall probably have occasion to refer to, in considering the diseases to which they are liable.

The general features of the Vale of Severn, although in some respects approaching to those of the Land's End, in others differ very considerably. That part of which the city of Worcester forms the centre is a midland district, extending the whole length of the county, from Bewdley on the north to near Tewkesbury on the south; a distance of about thirty miles. Its breadth, from the base of the Malvern chain to the somewhat elevated ground where the lias limestone appears, is about eight miles. The geological formation of the whole of this extensive valley, constituting the greater part of the plains of Worcestershire, belongs to the red marl, which is covered with a rich alluvial deposit, interspersed with numerous gravel-beds. The Malvern chain and outlines, which range from north to south, form the western boundary of this district, and are composed of rocks belonging to the granitic and transition series, with occasional appearances of basalt and other varieties of the trap rocks. The elevation of this chain varies from 1000 to 1500 feet above the level of the sea; that of the plains, bordering more immediately upon the Severn, is about eighty feet. The river Severn, which is usually confined low within its banks, runs through the centre of the district, in a direction nearly parallel with the Malvern Hills, and receives in its course the Stour, the Salwarp, the Terne, and other tributaries of less note; the Avon also joining it at Tewkesbury. The country, for miles round, is often flooded by the rise of the Severn and Terne, which are liable to overflow their banks whenever there are heavy and continued falls of rain in the upper country, whence they take their rise. Worcester, Stourport, Upton, and Bewdley, are the towns situated upon the banks of the Severn. Droitwich, which is also within the district, is on the Salwarp at no great distance. The water which rises through the red marl is, in many places, charged with sulphate of lime; the springs about Droitwich are more or less impregnated with salt, and those which have been sunk for the purposes of the salt-manufactory, long established in that place, are charged with it to the point of saturation. The Malvern waters are chiefly celebrated for their extreme purity. The climate of the Vale of Severn generally is mild and equable; a circumstance to which its proximity to a large arm of the sea, and its generally sheltered situation, materially contribute.* The number of rivers and the frequent inundations, with the low elevation of the greater part of the plains, necessarily render it humid; but the summers are warm, the vegetation luxuriant, at least in the marly districts, and the harvest some days earlier than in the neighbouring counties or in the Land's End. Of the natural productions of the Vale of Severn, the cider and perry afforded by the numerous apple and pear orchards, and the supply of hops from the extensive hop-yards, are the most important.

The chief occupations of the inhabitants are, in the country districts, agriculture; in the neighbourhood of Worcester, different branches of the glove manufacture and the china-works; at Droitwich and Stoke Prior, various operations carried on in connexion with the preparation of salt from the brine-springs of that neighbourhood. The general habits and condition of the poor present little that requires remark; the chief peculiarities being the frequent use of cider and perry, instead of malt liquor, in the agricultural districts; and the confinement and constrained position to which the females especially are subjected, from the nature of their occupation in the manufacturing of gloves. To some of the details connected with this subject we shall hereafter call attention, when considering their effects in the production of disease.

These observations, it will be remarked, chiefly apply to the more inland part of the district. It is, however, upon this that we have the most ample details, both in a topographical and a statistical point of view. The peculiar circumstances of Bristol, which is situated near the western extremity of the Vale of Severn, we shall draw attention to as occasion points out.

The difference in the longevity of the inhabitants of the two localities which we have thus rapidly surveyed, presents some features of considerable interest. Upon this point we have sufficiently full information in the tables published in the second volume of the Transactions. Table xiii. (*Trans. of the Prov. Med. and Surg. Assoc.*, vol. ii. p. 115,) shows the proportion of deaths to the living, in the whole county of Cornwall, taking the mean of the years 1811 and 1821, to be 1 in 66½; and that of the hundred of Penwith, or Land's End district, 1 in 64; that of the whole of England and Wales being 1 in 54. In Bristol, the ratio of mortality during ten years, from 1813 to 1822 inclusive, as ascertained by Drs. Carrick and Symonds, was 1 in 45. In the district lying between the river Severn and the top of the Malvern hills, an agricultural district forming part of the Vale of Severn, it is 1 in 65, as shown by Mr. Addison, from the returns of twelve years. From Table xv., however, (*Op. cit.* p. 119,) we learn that the ratio of mortality in the mining parishes of the hundred of Penwith (that is, in nine parishes in which the

* The mean temperature of Worcester, in the year 1832, was 52.7; the mean barometric pressure, 29.93. The mean temperature of Malvern, according to Mr. Addison, is 49.6.; the mean barometric pressure, 29.362; the mean range of the barometer, 1.155.

mining population, as compared to the other classes, is as 100 to 47,) is 1 in 58; whereas, in fifteen agricultural parishes, including towns and fishing villages, in which the mining population is to the other classes as 100 to 426, the ratio is only 1 in 64, or nearly the same as that of the Malvern district of the Vale of Severn. Dr. Hastings, however, states the rate of mortality in the whole county of Worcester at 1 in 52, and 1 in 53, as calculated upon the averages of five years preceding the several periods of 1811, 1821, and 1831, (*Illustrations of the Natural History of Worcestershire*, p. 17;) while from other documents, from an average of ten years, it appears to have been 1 in 56. The mean may be taken at 1 in 53. This ratio of mortality, however, includes the population of the towns; of which Worcester, containing about one-eighth of the whole population of the county, though by no means the most unhealthy, has a ratio of mortality of 1 in 48. These results will be more clear if exhibited in a tabular form.

| | Ratio of Mortality. | |
|---|---|---|
| In the whole of England | 1 in 54 | Mean of 1811 and 1821. |
| — whole county of Cornwall | 1 ... 66½ | Ditto. |
| — hundred of Penwith | 1 .. 64 | Ditto. |
| — hundred of Penwith | 1 ... 61 | 1811. |
| — mining parishes of ditto | 1 ... 58 | 1811. |
| — agricultural parishes of ditto | 1 ... 64 | 1811. |
| — whole county of Worcester | 1 ... 53 | Mean of 5 years, 1816 to 1820. |
| — city of Worcester | 1 ... 48 | 1821. |
| — agricultural district of Malvern | 1 ... 65 | Mean of 10 years, 1821 to 1830. |
| — city of Bristol | 1 ... 45* | Ditto of 10 years, 1813 to 1822. |

Now, by comparing the agricultural parts of each district, in which the occupation of the inhabitants is the same, and finding that the ratio of mortality of the one so nearly approaches to that of the other, we are entitled to infer, assuming these details to be correct, that the occupation of the population is that one condition which, of all others, would seem to influence most the average period to which life extends. Any differences which may exist between these two localities in climate, soil, natural productions, &c. appear either to be trifling in themselves, to counterbalance each other, or to exert but little effect on the general longevity of the population. The sketch which we have given of the respective topographies of the Land's End and Vale of Severn, however, shows that several important points of diversity are found in these situations, although those of climate have been perhaps over-rated. It remains, therefore, to be decided which of the two latter suppositions are correct. A very considerable difference, it will be observed, exists in the ratio of mortality of the cities of Worcester and Bristol, on the one hand, and that of the mining part of the Land's End, on the other. In the latter, which differs from the agricultural part of the same district chiefly in the occupation and mode of life consequent thereon of the greater portion of its inhabitants, we have a considerable increase in the rate of mortality; which is, however,

* These ratios are not quite in accordance with those deducible from the population returns of 1831; but we have thought it better to make use of the details afforded us in the several papers, as they seem to have been drawn up with great care by their respective authors, and entitled to the fullest credit.

still by no means equal to that of Bristol, or even of Worcester. In these situations, therefore, other causes, besides the occupations of their inhabitants, must come into operation; or these occupations must be presumed, taken conjointly, to be more injurious than mining. As we have before remarked, the diversities of climate have probably been over-estimated; and those of soil, or other conditions which are common to the agricultural and manufacturing or trading population, may, for various considerations, be thrown aside. Are the occupations, then, of these large towns, especially of Bristol, so extremely injurious in their effects upon the health and longevity of the working classes? or is the higher rate of mortality to be attributed, in great part, to the crowding together of the inhabitants, and to the more precarious or insufficient means of support in manufacturing and commercial towns?

The ratio of mortality of a district does not, however, indicate precisely the average longevity of its inhabitants, but is a compound result, arising from a consideration of the total population, increased by the births and influx of strangers, on the one hand, and diminished by the deaths and emigration, on the other; so that in a district in which there is a variable immigration of strangers, or emigration of its native population, the apparent ratio of mortality will be influenced by many circumstances which are foreign to the true ratio, or that which indicates the longevity of its native population. Further, the average longevity being deduced from the combined longevities of each individual residing in the district, it is evident that, from peculiar circumstances, acting at different periods of life, other elements than those simply expressive of the total numerical ratio should, in all cases, enter into the calculation, before we can deduce correct results as to the influence of any particular cause or causes operating in limiting the extent of the average longevity of the whole. The tables in the first part of the Essay on the Land's End District (Tables xv. to xxii., *Prov. Trans.*, vol. ii. p. 121 *et seq.*) are peculiarly instructive in regard to this point, as they not only show that, in the mining parishes of the hundred of Penwith, the longevity of the female part of the population greatly exceeds that of the male, and that, as compared with the agricultural parishes, the longevity of the agriculturist is greater than that of the miner; but that this difference obtains also in early life, and before the occupation could be supposed to exert any direct influence. The explanation of this fact is probably to be found in the more or less debilitated or diseased state of the parents, induced by their occupations or habits of life, operating in rendering their children of a more weakly constitution, even from birth.

To enter fully into such questions as these would require a much more extended notice than we can here devote to the subject, and we therefore turn to the consideration of the differences which may be found to exist in these several localities as respects the conditions of disease. The following table exhibits a numerical statement of the diseases occurring in the Land's End and at Worcester, classified and arranged for the purpose of convenient reference in the subsequent part of these observations.

| | Worc. city, 1832. | Worc. Disp. 1828—31. | Worc. Inf. 1828-30-32. | Worcester, Total. | Land's End. | Hobart Town. |
|---|---|---|---|---|---|---|
| 1. General fevers | 301 | 318 | 73 | 692 | 421 | 1097 |
| Eruptive fevers | 101 | 34 | 0 | 135 | 260 | 99 |
| 2. Rheumatism and neuralgia | 62 | 110 | 290 | 462 | 490 | 2428 |
| 3. Dropsies | 22 | 43 | 48 | 113 | 152 | 198 |
| 4. Scrofula | 26 | 21 | 55 | 102 | 253 | 93 |
| 5. Scirrhus and cancer | 2 | 5 | 20 | 27 | 42 | 4 |
| 6. Diseases of the brain and nerves | 94 | 133 | 180 | 407 | 638 | 1252 |
| 7. Diseases of the heart, &c. | 11 | 32 | 64 | 107 | 78 | 32 |
| 8. Diseases of the lungs: | | | | | | |
| Bronchitis, asthma, catarrh, &c. | 191 | 329 | 216 | 736 | 601 | 1784 |
| Phthisis and hæmoptysis | 112 | 235 | 69 | 416 | 297 | 202 |
| Pneumonia, pleuritis, &c. | 99 | 145 | 220 | 464 | 572 | 516 |
| 9. Diseases of abdomen: | | | | | | |
| Diarrhœa, cholera, and dysentery | 636 | 78 | 27 | 741 | 354 | 2711 |
| Dyspepsia, &c | 170 | 318 | 233 | 721 | 952 | 685 |
| Hepatitis and jaundice | 24 | 74 | 187 | 285 | 89 | 310 |
| Gastritis, peritonitis, and others | 69 | 101 | 88 | 258 | 630 | 2557 |
| 10. Diseases of the urinary organs | 10 | 24 | 16 | 50 | 106 | 118 |
| Diabetes | 0 | 2 | 8 | 10 | 8 | 0 |
| 11. Uterine diseases | 33 | 77 | 143 | 253 | 625 | 108 |
| 12. Chronic eruptions | 57 | 39 | 135 | 231 | 528 | 1457 |
| 13. Diseases of the organs of the senses | 46 | 42 | 176 | 264 | 293 | 1065 |
| 14. Syphilitic diseases | 38 | 34 | 115 | 187 | 51 | 1135 |
| 15. Bronchocele | 7 | 11 | 29 | 47 | 7 | 0 |
| 16. Miscellaneous diseases, chiefly surgical | 159 | 203 | 1132 | 1494 | 1344 | 6779 |
| Hernia | 5 | 9 | 256 | 270 | 36 | 363 |
| Accidents | 63 | 35 | 685 | 783 | 0 | 5015 |
| Totals | 2338 | 2452 | 4465 | 9255 | 8827 | 30008 |

We have been at some pains to draw up and arrange the preceding table from tables of diseases given in the Provincial Transactions and in the Midland Reporter. The results of the first column are obtained from the tables of disease as it appeared in the city of Worcester, during the year 1832, (*Transactions of the Prov. Association*, vol. i.); the second column is drawn up from the Reports of the Worcester Dispensary, for the years 1828, 29, 30, and 31, published in the Midland Reporter; the third from the reports of the Worcester Infirmary, for the years 1828, 29, 30, and 32,* (*Midland Medical Reporter*, vols. i. ii. and iii.; *Prov. Trans.*, vol. i. p. 373); the fourth presents the totals of the three preceding columns; the fifth column contains the results of Dr. Forbes's tables of disease in the Land's End district, and embraces a period of seventeen

* We should gladly have availed ourselves of the tables of disease given in the Reports of the Worcester Infirmary, for the years 1835 and 1836, and published in the fifth volume of the Transactions; but as fever, and other diseases presumed to be contagious, would seem to be excluded from that institution, and as we have no reports from the Dispensary, or from any other institution existing in that city at which such diseases come under treatment, we have thought it better not to include the results of these tables in the general aggregate here given.

years,—namely, three years, from 1810 to 1812, three years, from 1819 to 1821, and eleven years, from 1823 to 1833 inclusive, (*Provincial Transactions*, vol. iv. p. 167); and the sixth presents a general abstract of the returns, furnished by Mr. Scott, of the Medical and Surgical Diseases treated at the Colonial Hospital, Hobart Town, Van Diemen's Land, during the years 1821 to 1831 inclusive, (*Prov. Trans.*, vol. iii.,) and is here added for the sake of comparison with the results afforded by the preceding columns. We might have embraced with advantage the diseases of many other localities in this tabular view, especially those of London, of Carlisle, and of Birmingham, and might thus have been enabled to compare the conditions of disease among the vast and crowded population of the metropolis, and in large towns situated in the northern and central portions of the kingdom; but we are desirous, on the present occasion, of confining our attention as much as possible to a comparison between the two districts to which the preceding tables chiefly refer. We shall, however, have occasion, in the course of our observations, to refer to the state of disease as it is found to prevail in these and other places, with the view of illustrating the peculiarities of the districts more especially under consideration.

*Fevers.* General fever appears to be much more prevalent in the Vale of Severn than in the Land's End. The proportion in the city of Worcester, as obtained from the first and second columns of the preceding table,—by far the greater proportion of cases admitted at the dispensary, to which the second column refers, being from the city itself, (*Midland Reporter*, vols. i. ii. and iii.,)—is between one-seventh and one-eighth of the total cases of disease. Of the prevalence of fever in the surrounding country we are unable to form an estimate, as we have no returns of any fever-hospital, and the small number admitted at the infirmary, amounting only to seventy-three cases during a period of four years, out of 4465 cases of disease in general, shows that there is probably some restriction placed upon the admission of this description of cases within the walls of that institution; a restriction which would seem also to extend to other disorders presumed to be contagious, as we find no notice of any eruptive fevers having been admitted during the period referred to.* Out of 578 cases reported by Mr. Addison, (*Prov. Trans.*, vol. iv. p. 87,) it appears that 64, or 1 in 9, were fever; so that the country lying between the banks of the Severn and the base of the Malvern Hills, including also the elevated village of Malvern itself, would not seem to enjoy much greater immunity from the causes generating fever than the more crowded and confined districts of the city of Worcester. This proportion, namely 1 in 9, is, however, somewhat too high, as Mr. Addison has not included surgical cases in his return. Still we may fairly draw the inference that the fever of the Vale of Severn, prevalent during the period to which these observations refer, is to be attributed chiefly, if not altogether, to the operation of causes in a great measure independent of the artificial habits or condition of the population, and partaking either of the nature of emanations from the soil, or

* In the returns of the Worcester Infirmary for 1835 and 1836, out of 2627 cases of disease, 36, or 1 in 73, were cases of continued fever, which approximates very closely to the results of the preceding years.

of some atmospherical condition, or (to use the language of Sydenham) reigning epidemic constitution, operating more or less throughout the whole district. We are strengthened in the former of these opinions by observing that, in those parts of the district referred to in which miasmatic exhalations more especially prevail, the cases of fever were of more frequent occurrence; as in the low lands bordering the river Severn, and particularly among the population living within the sphere of influence of a canal which skirts one of the suburbs of the city of Worcester, (*Midland Reporter*, vol. i. pp. 207 and 349.) In Bristol, the number of fever-cases, in 700 of disease generally, admitted at the infirmary during the year 1832, as stated by Drs. Carrick and Symonds, (*Prov. Trans.*, vol. ii. p. 177,) was 171, or nearly one-fourth. How far this very high ratio of prevalence is to be attributed to a more crowded and more necessitous population, or to the peculiar circumstances under which this city is placed with respect to the accumulation of refuse of every description in the muddy bed of the Avon, the exhalations from which are, from the rise and fall of the tide, periodically left to spread their deleterious influence over the crowded and ill-ventilated streets of the lower town, is a question for the decision of those who are better acquainted with the locality; but the following extract from the essay of Drs. Carrick and Symonds on the Medical Topography of Bristol, published in the second volume of the Transactions, is to us a sufficient ground for attributing the prevalence of fever to the latter of these causes, although we doubt not that its severity may be materially increased by other circumstances.

"Before the floating harbour was constructed, every facility was afforded for the removal of filth, by the unobstructed course of the rivers; [the Frome and Avon, upon the junction of which Bristol is situated;] but, after the accomplishment of that work, the accumulation of the matters discharged by the drains in a stagnant body of water occasioned no slight inconvenience to the citizens for many years." . . . . "Within the last few years, it has been customary to empty the float twice annually, and a large sewer has been formed, which runs from the embouchure of the Frome in a direction parallel with the float, and, after receiving the drains from the central parts of the city, discharges itself into the new channel of the Avon. Into this sewer the Frome sends a division of its stream, loaded with the contributions which it has received at every step of its progress through some of the most closely built and densely crowded districts. Unhappily the current of this river is narrow, torpid, and scanty; in consequence of which it often struggles ineffectually with the burthens accumulated upon it, and deposits them upon its bed, the sides of which become elevated into pillows for the exhausted, almost stagnant, waters, and exhale miasms sufficient, it might be imagined, to infect the whole neighbourhood. In the summer time we have seen the stream shrunk to a width scarcely exceeding that of a large gutter, and trickling between two mounds of mud. At all times it is almost impossible to cross the bridges by which it is concealed from sight, in the midst of streets and lanes, without being reminded, by particular odours, of its propinquity." (*Prov. Trans.* vol. ii. p. 162.)

The superiority of the Land's End in this respect is very great; the proportion of fever cases to those of other diseases being scarcely more than 1 in 21; evidencing, in a remarkable manner, either the absence of miasms, at least such as are capable of generating fever, or their speedy dispersion, or neutralization by other agents in operation throughout the district. It is a curious fact, and one which tends much to confirm the accounts which have been given of the salubrious nature of its climate, that, in the newly broken-up country of Van Diemen's Land, the proportion of fever should be so low as 1 in 27; or, excluding the accidents, the number of which is very great, 1 in 23. Out of 18,663 cases received as out-patients at the Birmingham Infirmary, during the years 1832 to 1835 inclusive, (*Reports of the Out Cases attended at the Birmingham Infirmary*, by Messrs. Parsons and Ryland, *Provincial Transactions*, vols. i. to iv.), there were 1468 cases of fever, or 1 in 12.7. This is a ratio of prevalence considerably lower than those of the Vale of Severn, which we have previously given. At Plymouth, according to Dr. Blackmore's Tables (*Reports on the Diseases of Plymouth*, by E. Blackmore, M.D., *Edinburgh Med. and Surg. Journal*, vol. xxxi. p. 269), of 5648 cases there were 400 of fever, or 1 in 14.2; a ratio lower than that of the midland town of Birmingham, but still considerably higher than that of the Land's End. It would hence appear that the opinion expressed in the Essay on the Medical Topography of the Land's End, as to "the great prevalence of the common continued epidemic fever of this country, variously denominated contagious fever, typhus, &c. in the town and immediate vicinity of Penzance," (*Prov. Trans.*, vol. iv. p. 162,) however just in itself, is by no means applicable to the district in question, when compared with other localities. The total number of cases of general fever included in the Penzance Tables, from which the ratio before given, namely 1 in 21, has been deduced, is 421; but in this number are included 26 cases of infantile remittent, and 10 of hectic. Excluding these, and taking the cases of continued fever only, the common epidemic of the country, with a very few, not more than three cases of intermittent, we have 385 cases extending over a period of seventeen years; that is between twenty-one and twenty-two cases of fever each year, which, as compared with other places, seems to be a small number to apply for relief from this cause at a public institution in the midst of a crowded population. At Bolton le Moors, in which continued fevers ("typhus and low fevers,") are thought, of late years at least, not to have prevailed to any serious extent (*Medical Topography of Bolton*, by Dr. James Black; *Prov. Trans.*, vol. v. p. 201,) of 1901 cases of disease occurring between the 1st of November, 1824, and the 31st of October, 1825, 68, or 1 in 28, were synochus and typhus; which does not differ greatly from the Land's End ratio of the same forms of febrile disease. The far greater prevalence of common continued fever in the cities of Worcester and Bristol, situated as they are in the midst of malarious districts, or at least of low, moist meadows on the banks of rivers, and exposed to the exhalations arising from stagnant waters and canals, is a strong evidence in favour of the views advocated by the late Dr. M'Culloch; and the testimony of the older practitioners in Cornwall, who are of opinion that "sporadic fevers of a continued or imperfectly remittent character, and of a contagious nature, were more frequently met with in former years, more particularly in the lowest and dampest localities, and on the more uncultivated moors," (*Prov. Trans.*, vol. iv. p. 165,) is to a certain extent confirmatory of the same opinions. It will be observed that these remarks are somewhat at variance with the opinion expressed in the Essay on the Medical Topography of the Land's End.

"In explanation of the cause of the continued prevalence of epidemic fever in this district, it will, I apprehend, be considered sufficient, by most persons, to refer to the economical details in the former part of this paper. In the close, ill-ventilated, and dirty tenements, in the poverty and deficiency of food and clothing, and in the mental depression consequent thereon, we may find ample causes of fever, although scarcely any of the usual sources of malarious influence are to be traced in the district—a fact still more strikingly demonstrated by the almost total absence of intermitting and remitting fevers. This is a circumstance deserving the attention of those who are disposed, with the late Dr. Armstrong, to look to terrestrial miasmata for the cause of all our fevers," (p. 165.)

There can be little doubt that the views of Drs. M'Culloch and Armstrong were too confined and too exclusive upon this point; but the absence of intermitting or remitting fevers by no means proves the absence of malaria. Malarious influence may be present, although not in such intensity as to develop the purely intermittent or remittent types of fever; and, in the present state of our knowledge, it is impossible to say how far the slighter degrees of this influence, or the modified sources from which it is derived, may operate in the production of the *more* continued form which fever has assumed, not only in this locality, but throughout the country generally, since draining has been more extensively and more regularly practised. We have said the *more* continued form, because we believe that few who have been accustomed to observe the common epidemic fever of this country will deny that, in the earlier stages, at least, and even after the disease is fully formed, an evident tendency towards remission occurs in almost every case, the exceptions being commonly found only in close ill-ventilated apartments in confined situations.

There appears to be little difference in the general characters of the fever in the two districts more especially under notice. Dr. Forbes states, that "the continued fever of the Land's End exhibited nothing peculiar in its character. It was often very severe, long-protracted, and frequently fatal. Death occurred rarely from sudden collapse; sometimes from intestinal hemorrhage; commonly by the typhoid or comatose state, indicating an affection of the brain of an unknown quality; never by the supervention of anything like putridity," (p. 164.) The following extract from the Report of the Diseases of Worcester during the year 1832, by Dr. Streeten, shows the character of the disease in that situation:

"The fevers which occur in Worcester, necessarily vary according to the dryness or moisture, and other changes of the atmospheric condition, and according to the greater or less exposure of the particular localities in which these affections are prevalent to the operation of such changes; but as those parts of the town which are chiefly inhabited by the poor, amongst whom fever is generally found to prevail more than in the more elevated classes of society, are low in situation, and exposed to exhalations arising from the presence of moisture and a mixture of vegetable and animal refuse, the fever which occurs here, is chiefly of such a nature as that which arises in corresponding situations elsewhere. Intermittents in their most distinctive form are scarcely known, for there are no purely marshy or fenny spots; but from the effects of imperfect drainage, and the mixture of animal and vegetable exhalations, fever of a mild typhoid type is very common,—many parts of the town, and particularly those situated in the ill-drained districts bordering upon the canal, being scarcely ever entirely free from it. In a general sense Worcester may be said to be tolerably well ventilated; and, accordingly, true typhus fever is of comparatively rare occurrence, though instances of apparent communication of the usual low typhoid fever from one individual to another, are occasionally met with. The local affections with which these fevers are commonly accompanied, are most frequently a sub-acute bronchial inflammation, which is especially found on the banks of the river, or in those who are, by their occupations, predisposed to pulmonary affections of this description. In other parts of the town, and especially in such as are damp and ill drained, as is the case with the localities before referred to as bordering on the canal, and in some low grounds near the river, a gastro-enteric affection is a frequent accompaniment, marked by more or less pain at the pit of the stomach, tenderness of the abdomen, diarrhœa, and, sometimes, even hemorrhage from the bowels. Severe headach, with delirium and general marks of inflammatory action within the cranium, are chiefly found in the suburbs and more open districts, and conjoined with greater depression of the powers of the nervous system, in the close and badly ventilated spots in the heart of the city." (*Prov. Trans.*, vol. i. p. 380.)

The following observations by Dr. Hastings in his Illustration of the Natural History of Worcestershire are also well worthy of notice. "The whole of this county," he remarks, "in which the sandstone prevails, enjoys a great freedom from severe febrile diseases, the worst forms of typhus fever being rarely witnessed; and, from my own experience, I may assert that the hilly parts of Worcestershire, where there is a clay soil, as about Broadway, and the line of hills running in that direction, are much more prone to be affected with fever than the sandstone district which comprises the lower parts of the county. This immunity from severe forms of fever is not confined to the rural population; but is in a great measure also to be observed in the large towns. In Worcester it is a very rare thing to meet with very severe cases of fever, and the worst forms of the disease in this town are seen after flood-time in the vicinity of the river Severn," (p. 31.)

But although the general characters of fever, as it occurs in Worcester and the Land's End, present but few distinctive marks, it would appear that, in the latter situation, the cases were on the whole more severe, and consequently more fatal. The ratio of mortality from this disease is not given in the Penzance Tables, but in Worcester it is very low; as of 306 cases occurring at the Worcester Dispensary, from 1828 to 1831, it is stated that only 13, or 1 in 23½, proved fatal; and of 96 occurring at the same institution during the year 1832, there were four deaths, or 1 in 24, almost exactly the same proportion as the average of the preceding years. (*Prov. Trans.*, vol. ii. p. 384.) Dr. Blackmore, gives as the ratio of mortality of fever cases in Plymouth, 1 in 21, which does not greatly differ from that of Worcester; while in Edinburgh, according to Dr. Craigie's Clinical Report, of 825 cases of fever admitted at the Infirmary, from the 25th of October, 1834, to the 24th of October, 1835, 83, or 1 in 9½, proved fatal. (*Edin. Med. and Surg. Journ.*, vol. xlvi. p. 37.) 1 in 15.6 is given by Mr. Parsons as the ratio of the years 1833, 34, and 35, at Birmingham. (*Prov. Trans.*, vol. iv. 490.) In the London Fever Hospital, according to Dr. Southwood Smith, the proportion of deaths is very much higher, amounting to about one-fourth of the cases.

The tables afford but little information respecting eruptive fevers, under which are included Small-Pox, Measles, Scarlet-Fever, Urticaria, and Erysipelas; as the number of cases is not great, and from the greater proportion of such cases being usually the subject of domestic treatment, those which are included in Infirmary or Dispensary Returns afford but little indication of the extent to which they frequently prevail. It should however be remarked, that the ninety-nine cases mentioned in the Van Diemen's Land returns were all cases of erysipelas; none of the epidemic eruptions having as yet made their appearance in the colony.

*Rheumatic and Neuralgic Diseases.* In the Penzance Tables, the various forms of rheumatism and neuralgia are classed together under one general head, and amount to 490 cases in all. Of these, 439 were acute and chronic rheumatism, and 51 sciatica, lumbago, neuralgia, and nervous pains. The proportion to the whole number of cases is 1 in 17.6, but there appears to have been a considerable variation in the degree of prevalence of this class of diseases in different years embraced within the period over which the reports extend, and a remarkable increase during the later years. The following is the proportion of rheumatism, as observed in various localities:

| | | |
|---|---|---|
| Worcester city . . | 1 in 30.6 | Dispensary Reports, Midland Reporter. |
| Birmingham . . | 1 ... 29.0 | Messrs. Parsons and Ryland, Prov. Transactions. |
| North of England . | 1 ... 22.0 | Dr. Haygarth, quoted by Dr. Forbes. |
| Malvern* . . . | 1 ... 20.6 | Mr. Addison, Prov. Transactions, vol. iv. p. 136. |
| Penzance . . . | 1 ... 20.3 | Dr. Forbes, Prov. Trans. vol. iv. p. 177. |
| Worcester city & county† | 1 ... 20.0 | Midland Reporter, Prov. Trans. (see Table.) |
| Plymouth . . . | 1 ... 18.4 | Dr. Blackmore, Ed. Med. Journ. vol. xxxi. p. 271. |
| Penzance‡ . . . | 1 .. 17.6 | Penzance Tables, Prov. Trans. vol. iv. |
| Bolton le Moors . | 1 ... 17.6 | Dr. Black, Ed. Med. Journal, vol. xxv. p. 444. |
| Plymouth . . . | 1 .. 17.0 | Dr. Woolcombe, quoted by Dr. Forbes. |
| London . . . | 1 .. 14.7 | Drs. Willan and Bateman, quoted by Dr. Forbes. |
| Van Diemen's Land . | 1 ... 12.3 | Mr. Scott, Prov. Trans. vol. iii. |
| Bristol§ . . . | 1 ... 6.8 | Drs. Carrick and Symonds, Prov. Tra. vol. ii. p. 177. |

It is obvious, from a consideration of the preceding results, that one of the causes upon which rheumatism has been supposed to depend is not of that importance in the production of the disease which has been assigned to it. Of the ten localities given in the preceding table, the highest ratios of prevalence are by no means to be found in the coldest situations. Thus, in the north of England, the proportion of rheumatism is only 1 in 22; whereas, in the milder climates of the Land's End, Plymouth, and London, it is respectively 1 in 20.3, 1 in 17, and 1 in 14.7. The influence of moisture cannot, however, fail to be noticed in the cases of Plymouth and London, and more especially Bristol. In this last-mentioned site, the ratio of prevalence is as high as 1 in 6.8; and though, as we have remarked, this is by no means to be taken as the actual proportion in which the disease occurs, still its frequency is such as considerably to exceed most other places in the kingdom. This, however, is not difficult to account for, situated as Bristol is, on the Western side of the island, near the mouth of a large river, or rather arm of the sea, and upon the junction of two smaller rivers, with floats and basins and stagnant waters intersecting the town; the damp exhalations arising from which must necessarily be much confined in the lower portions of the town by the surrounding elevated land.

The greater prevalence of those causes which are instrumental in the production of neuralgic disorders in the Vale of Severn than in the Land's End, is shown by the relative increase in the ratio of prevalence in

* This probably includes lumbago, sciatica, and perhaps neuralgia.

† Lumbago, sciatica, and neuralgia are included in this ratio. ‡ Ditto.

§ This is a very high ratio, and is obtained from 700 medical cases treated in the Bristol Infirmary, of which 117 were rheumatic. Making every allowance for the addition of cases of lumbago and sciatica, which are probably included in the 117, and the exclusion from the total of the surgical cases, it will still much exceed the ratio of other places.

the former of these localities, when this class of diseases are taken into the estimate. Thus, in Worcester, the ratio of prevalence of rheumatism alone is 1 in 30.6, which, by including the neuralgic affections, is increased as much as to 1 in 20; whereas, in Penzance, where the proportion of rheumatic cases is 1 in 20.3, the increase from the addition of neuralgia is not more than to 1 in 17.6.

It may be observed, that this mode of computing relative proportional results, by comparing the increase of the ratios of prevalence which is obtained by the addition of other elements, although not affording conclusions absolutely correct, is yet of great importance in some cases, as giving a closer approximation to the truth than can be attained by the ordinary method of comparing the apparent ratios of prevalence of any one disease, taken separately. Many causes tend to affect the correctness of the proportions thus ascertained; as, the different views of the authors by whom the diseases have been respectively classified; the admission or omission of surgical diseases, and of the slighter medical cases in the tabular arrangements, &c. Thus, in the Hobart Town Report, the proportion of surgical cases is very large; the accidents alone amounting to rather more than one-sixth of the whole number of cases registered; and, though we might easily get rid of this source of error by omitting this class of affections from the estimate, which indeed, strictly speaking, should be done, (as accidents have nothing to do with the general conditions of disease,) we are precluded from doing so on the present occasion by some of the tables enumerating these among other surgical diseases. But the relative increase in the ratio of prevalence of a disease, in one locality as compared with another, by the addition of another disease, incontestibly shows that, in the two localities, one class of cases is proportionally of more frequent occurrence than the others; as in the instance above given of rheumatism and neuralgia. Setting aside other sources of error, we are thus fully entitled to conclude that neuralgic affections, as compared with rheumatism, are relatively more prevalent in Worcester than in Penzance; or, in other words, that the causes generating neuralgic affections, whether really more prevalent in Worcester or not than the causes generating rheumatism, are relatively in greater activity than at Penzance, although in this latter situation both the one and the other may be actually of greater or less prevalence.

The proportion of cases of acute rheumatism to those of the chronic form of the disease, appears to vary greatly in different parts of the kingdom. Dr. Forbes regards the acute disease as of comparatively rare occurrence in Cornwall.

"The whole cases seen by me, in four years and a half, were four in number, viz. two in 1817, and two in 1821; not one case having been met with in the three intermediate years. In the Dispensary Report for 1821, I stated, 'During the whole of the last two years, not one case of acute rheumatism has been entered in the books; and I cannot help thinking that the total absence of the disease, among so large a body of individuals, for so long a period, is a circumstance that would be reckoned very singular in the northern or even central parts of our island. It is, at all events, I think, sufficient to render doubtful the truth of a common notion, that a moist climate is favorable to the production of rheumatism." (*Prov. Trans.*, vol. iv. p. 174.)

It appears, however, from the testimony of Dr. Montgomery, to have been of more frequent occurrence at Penzance in subsequent years. The numerical proportion of the acute to the chronic disease is stated by Dr. Forbes to have been, during his residence, and the two years subsequent to his leaving the Penzance Dispensary, 5 of the former to 69 of the latter, or nearly 1 in 14 (1 *to* 14, that is, 1 *in* 15.) The proportion in London appears to be 2 in 7, and the mean of the London and Plymouth tables, and Dr. Haygarth's Reports from the north of England, referred to by Dr. Forbes, is 1 in 3. The proportion, however, in the midland counties varies greatly from those here given. In the Reports of the Worcester Dispensary for the year 1828, (*Midland Medical Reporter*, vol. i.), the distinction between the acute and chronic forms is observed, and out of 20 cases there were 12 of the former to 8 of the latter, or 3 in 5. In the year 1835, of 75 cases admitted at the Worcester Infirmary 54 were acute and 21 chronic, or nearly 3 to 1, (*Prov. Trans.*, vol. v.) In the Birmingham tables (*Prov. Trans.*,) of 642 cases, 416 were acute and 226 chronic, being nearly 2 in 3, or in the proportion of 2 to 1; exactly reversing the mean of London, Plymouth, and Carlisle. From Mr. Watson's observations on the Medical Topography of Stourport (*Prov. Trans.*, vol. ii. p. 181), it may also be inferred that acute rheumatism is of frequent occurrence in that neighbourhood, while the chronic form of the disease is not so: he says, "I have never considered chronic rheumatism as a frequent disease here; but I must observe, that I have generally considered those pains which are commonly called rheumatic as depending upon a disordered state of the digestive organs, and treated them as such," (p. 197.)

The causes of this great variation in the relative prevalence of the acute and chronic forms of rheumatism in different localities do not appear; and it will probably require an extended series of observations to remove the obscurity in which they are at present involved.

"I am not aware," (says Mr. Watson,) "that it is more frequent among watermen than among those persons who are less exposed to wet and cold; and at least one half of my patients have been young women. Nor are those persons who are exposed to great change of temperature in foundries and forges more liable to rheumatism: I do not, at present, recollect a single case of acute rheumatism among them." (*Prov. Trans.*, vol. ii. p. 196.)

"The evidence supplied by the practitioners of the district," says Dr. Forbes, "as to the relative prevalence of rheumatism among the different classes of the community is somewhat contradictory, but the weight of testimony is decidedly in favour of the opinion that the disease is not more prevalent among miners than among other labourers; and this is the conclusion to which my own observation leads. It must be owned, that this is rather a singular circumstance, according to the commonly received etiology of the disease, when we refer to what was stated in a former part of this paper respecting the extreme dampness, or rather wetness, of some of the mines. Would it, therefore, seem probable that the effect of the moisture was counteracted by the warmth of the locality, and the immunity from currents of air in the bottom of the mines?" (*Prov. Trans.*, vol. iv. p. 177.)

These remarks, however, refer to the chronic as well as to the acute form of the disease, and apply rather to the occupation of a portion of the population than to the circumstances of the whole district. The suggestion thrown out as to the probable influence of the warmth of the mines, in counteracting the effects of the damp, is equally applicable to those who work in foundries and forges, alluded to by Mr. Watson: but in these latter situations there is certainly no immunity from currents of air:

possibly in both instances the continued and violent exercise, and the consequent open state of the skin, may act as a counteracting influence. However this may be, we must observe that our own experience does not bear out Mr. Watson's statement, that acute rheumatism is not more frequent among watermen than among persons less exposed to wet and cold; and that, with respect to foundries and forges, humidity, which we are inclined to consider as the more efficient cause in the production of the disease, does not particularly prevail.

We have dwelt thus long upon the subject of rheumatism, because we believe it to be one upon which erroneous opinions are very prevalent; at least the commonly received views as to the causes of the disease would seem to have been taken up without a sufficient examination of the grounds upon which they have been adopted. Nothing is wanting to develop the true bearing of the alleged causes, cold and humidity, in the production of the disease, but a series of careful observations in different parts of the country. There are few diseases which exert a more baneful influence upon the welfare and comfort of a considerable portion of the community. Not only is the attack itself frequently of extreme severity, and, from the protracted duration of the symptoms, attended with considerable loss of time to those who can ill spare it, but the foundation of permanent derangement of health, especially amongst the labouring part of the community, is too frequently laid in permanent and irretrievable organic disease of the heart, or in thickening and contraction, or other changes, of structure in the joints. The real remedy for these serious evils is to be found in the adoption of such measures as are calculated to prevent, or at least to diminish, the frequency of its occurrence; and efficient measures can only be devised for the attainment of this purpose from an accurate acquaintance with the true cause or causes of the disease. It is not, therefore, too much to require from those who are placed in circumstances which enable them to enter upon the enquiry with advantage, such a series of observations; and, if these are made with the requisite care and attention to particulars which all such enquiries demand, there can be no doubt but that valuable knowledge will be obtained.

*Dropsies.* Medical statistics are as closely connected with, and as capable of throwing light upon moral statistics, as other branches of statistical enquiry. They may, indeed, be frequently regarded as another mode in which the effects resulting from the operation of the various circumstances acting on population are expressed. Assuming our knowledge to be in a sufficiently forward state for the purpose, the prevalence of certain diseases tends to show the circumstances of the locality in which they occur; and, again, the knowledge of the circumstances of the locality affords an equal indication as to the conditions of disease which prevail therein. As a familiar illustration of this principle, we may refer to the existence of intermittents in marshy countries. The return of numerous cases of ague in a series of medical reports necessarily leads to the inference that the locality to which these reports refer contains a considerable portion of marshy land; while, on the other hand, if, in a topographical account, we are informed that the district is low, marshy, and abounding in stagnant waters, we equally infer that some forms of intermittent or remittent disease are necessarily prevalent there. The application, however, of this principle must, in the present state of our knowledge, be made with great caution. Perhaps there is no class of affections with which we are acquainted which has been considered to develop the moral state of a locality in which they are found to occur more than dropsies. The prevalence, for a series of years, of the various forms of dropsy has been thought to be a pretty sure indication that the population of the district are more or less given to intemperance, especially in the use of ardent spirits. Guided by this principle, we should perhaps infer, from the proportion of dropsical cases, that such was the case in the Land's End, the ratio of prevalence of dropsy in this locality being as high as 1 in 58; but Dr. Forbes observes, that both the mining and agricultural population, notwithstanding the statements of Polwhele and Dr. Borlase as to their former intemperate habits, "must now be considered as habitually sober, although," he adds, "they, unquestionably, occasionally commit excesses in this particular." (*Prov. Trans.*, vol. ii. p. 101.) This opinion, however, would seem to admit of some limitation, from what is stated in the continuation of this essay. "Dropsy," it is remarked, "appeared not to affect miners, or any one class of persons more than another; unless indeed we reckon *drunkards* as a class, and as the chief of them the landlords of public-houses; the greater number of whom here, as elsewhere, fall victims to their intemperance, of which some form of dropsy is the most obvious result; although itself generally a consequence of some other disease. The frequency of dropsy in this district seems also established by the mortuary registers both of St. Paul and St. Hilary, particularly of the former, in which it bears a proportion of no less than one in twenty of the total deaths." (*Prov. Trans.*, vol. iv. p. 201.)

The ratio of prevalence of dropsy in the Land's End is, as we have already stated, 1 in 58; in London, it is probably nearly the same; in Worcester, it is 1 in 82; in Bolton le Moors, 1 in 79; in Plymouth, again, where probably, from the number of sailors, there is a considerable consumption of spirits, it is 1 in 55; and in Bristol, also a sea-port town, it is 1 in 32. In Birmingham, the ratio is as low as 1 in 155; but, as the reports of Messrs. Parsons and Ryland refer only to the out-patients of the Infirmary, and dropsy is generally a very severe disease, and therefore more likely to be received within the walls of the institution, we can scarcely take this as affording even an approximation to a correct result. The low proportion in Worcester is more likely to be owing, at least in part, to the substitution of cyder, perry, or ale, for spirits, than to any superior morality of the population, though it is also probable that the manufactory of that city and its immediate vicinity, not requiring much expenditure of physical force, may also have a negative effect upon the habits of the people, in rendering them less inclined to excess in the use of intoxicating liquors than occupations which are more laborious. Anasarca appears to be decidedly the most frequent form of dropsical disease. Of 87 cases occurring at the Penzance Dispensary, during eight years of the seventeen over which the returns extend, 62 were cases of anasarca, 22 of ascites, and 3 of hydrothorax. Dr. Forbes is disposed to consider the proportion of anasarca as unusually large; and the results of the Plymouth Tables, furnished by Dr. Blackmore, tend to confirm the correctness of this opinion. Of 102 cases occurring at Plymouth, 56 were anasarca, 31 ascites, and 15 hydrothorax. In the Worcester

Reports, of the 65 cases entered in columns 1 and 2, 29 are recorded as anasarca, 12 as ascites, and 13 hydrothorax; the remainder being simply designated hydrops, or general dropsy. Of 57 cases included in the reports of the Worcester Infirmary for the years 1835, 1836, (*Prov. Trans.*, vol. v.), 26 are anasarca, 27 ascites, 3 hydrothorax, and 1 general dropsy; giving, of 110 cases occurring at Worcester classed under the heads of anasarca, ascites, and hydrothorax, 55 of the first or exactly one half, 39 of the second, and 16 of the third form of the disease. The following arrangement of these results exhibits the proportionate numbers of the different forms of dropsy in one hundred cases of the disease at the several localities referred to:

| | Land's End. | Plymouth. | Worcester. | Mean. |
|---|---|---|---|---|
| Anasarca . . . | 71.3 | 53.7 | 50.0 | 58.3 |
| Ascites . . . | 25.3 | 22.2 | 35.5 | 27.7 |
| Hydrothorax . . | 3.4 | 24.1 | 14.5 | 14.0 |

We cannot help adverting to the remark made by Dr. Forbes, in a preceding quotation, in regard to the liability of *Innkeepers* to dropsy, as we have always regarded the fate of this class of men as one of the most striking illustrations of the destructive influence of intemperance. During a long course of practice, we have been struck with the speedy and almost regular disappearance of one landlord after another from nearly all the smaller inns of an extensive district, including a large town and country population. All of these men, and most of them in the prime of life, have fallen victims to diseases originating in drinking; the temptation to this vice being almost always found too strong to be resisted by persons of this rank of life, when perpetually presented to them. We doubt not but this statement will be confirmed by all our provincial readers of much experience.

*Scrofula.* The proportion of glandular diseases appears at first sight to be much greater in the Land's End, where the ratio of prevalence is as high as 1 in 35, than in the midland counties, where it is no more than 1 in 90; but there is a source of fallacy here, which, although it prevents the attainment of any accurate conclusion upon the subject, it is necessary to take note of, lest the inspection of the tabular results before given should lead into error. In the Land's End Tables the section, *External Scrofula*, comprises 253 cases, including rickets, enlarged glands, diseased joints, and cutaneous ulcers; whereas, in the Worcester Tables, by the term *Scrofula* it is simply indurated, enlarged, and ulcerated glands which seem to be intended; amounting to 102 in the whole. In the third column, among the miscellaneous surgical cases, are included fifty-six cases of diseased joints, many of which were probably scrofulous, although no indication of their being of this nature is given in the Reports of the Worcester Infirmary, as published in the Midland Reporter, from which this column has been drawn up. Still the proportion of external scrofula is evidently greater in the Land's End district, a circumstance which may perhaps be in part accounted for by the greater prevalence of internal tubercular disease in the Vale of Severn.

"It appeared to me," observes Dr. Forbes, "that the common external indication of the scrofulous constitution, visible in the complexion, texture of the hair, configuration of the body generally, and of the features of the face more particularly, were more marked among the inhabitants generally, and especially among the upper classes, than in other parts of England, where I have had opportunities of observing the condition of the people; and I thought I could discover a larger proportional number of white swellings, incurvations of the spine, hip cases, and glandular tumours, there than elsewhere. Of the very general and great prevalence of these diseases throughout the district, the answers of the resident practitioners, under the heads *scrofula*, *rickets*, *white swelling*, *hip-disease*, *spine-disease*, are sufficient evidence; but then absolute proportional superiority in point of frequency, relatively to other districts, cannot be considered as established on the present evidence." (*Prov. Trans.*, vol. iv. p. 190.)

Dr. Forbes is inclined to attribute this prevalence of scrofulous disease, first, to the relaxing nature of the climate arising from its extreme humidity and equable temperature, and, secondly, to the frequent intermarriage of families, owing to the isolated character of this district.

"This latter circumstance," he continues, "is likely to operate in two ways; first, by generating the strumous diathesis in healthy subjects, on the well-known principle of breeding *in and in;* and, secondly, by tending to develop and strengthen the predisposition, when already existing. There is no fact better established in physiological pathology, than that the offspring of parents, both of whom are touched with any hereditary malady or morbid predisposition, are likely to exhibit the same malady or morbid predisposition in a greater degree than either of the parents." (p. 191.)

The low ratio both of external glandular disease and of internal tubercular disease (phthisis) in Van Diemen's Land, (that of the former being only 1 in 322, and of both together not exceeding 1 in 101,) is a curious and very interesting fact.

*Scirrhus and Cancer.* There are two circumstances attending the affections arranged under this head to which it will be right to direct attention, although the small number of cases recorded prevents our drawing any conclusions respecting them. The first of these is, their much greater prevalence in the Land's End than in Worcester; the proportions being, in the former case, 1 in 210; in the latter, 1 in 342: the other is, the almost entire immunity of Van Diemen's Land from affections of this nature; the number, in upwards of 30,000 cases, being only 4, or 1 in 7502.

*Diseases of the Brain and Nervous System.* These are much more prevalent in the Land's End than in the Vale of Severn; the total number in the former of these localities being 638, or 1 in 14.8; in the latter, 407, or 1 in 22.7. This section, however, embraces so many diseases differing in their nature, in the circumstances under which they are found to occur, in the classes of inhabitants which they severally affect, and in the causes to which they may be respectively attributed, that, without a more extended analysis of them than our limits will permit, it would be impossible to derive any advantage from their consideration.

*Diseases of the Heart and Blood-Vessels.* Considering the great exertions made by the miners when at work, and on their return from it in ascending the ladders to the surface with a considerable load upon their backs, and the prevalence of bronchitis, (especially the chronic forms,) of asthma, and of rheumatism, among them, we should have expected that organic disease of the heart would have proved of very frequent occurrence. The whole number of cases, however, reported in the Penzance Tables, amounts only to 78, affording a ratio of prevalence of 1 in 113; whereas, in Worcester, where the causes presumed to be influential in inducing changes of structure in the heart and larger blood-vessels do not seem to exist in such intensity, the number of cases returned in the various reports is 107, the ratio of prevalence 1 in 82. This difference, however, we are inclined to attribute, at least in part, to diseases of the heart being better understood and more readily recognized in the present day than formerly. The Worcester reports extend over a period of five years, from 1828 to 1832 inclusive: the Penzance Tables consist of the returns of three successive periods, the first two of which are three years each,—namely, from 1810 to 1812, and from 1819 to 1821, and the last eleven years from 1823 to 1833. In the first period only one case of diseased heart is entered, in the second period not more than four; so that in six years, which it should be observed were previous to the introduction of mediate auscultation, five cases only are recorded to have occurred; whereas, in the last period of eleven years, we have no less than seventy-three, or nearly the whole number. This statement accords with the opinions expressed by Dr. Forbes in the following passage:

"I have no remark to make on this class of diseases, except to express my belief, founded partly on reasoning and partly on my own observation, that miners are more subject to organic changes of this organ, particularly dilatation, than most other classes of workmen. The causes of such a morbid state, in this class of persons, stand out prominently, both in their habits and diseases. The extreme strain on the respiratory and circulatory organs produced in the ascent from mines, described in the first part of this paper, and the chronic obstructions to the transmission of blood through the lungs, so frequent a form of disease in this class of persons, are strikingly calculated to produce these affections." (*Prov. Trans.*, vol. iv. p. 203.)

The practice alluded to in the foregoing extract, as to the mode of ascending from the mines, will be found described at length in the preceding part of the essay, (*Prov. Trans.*, vol. ii. p. 95,) and its injurious effects upon the organs of respiration and circulation forcibly pointed out in the letters from Dr. Wise and other practitioners inserted towards the conclusion of the second part. (*Prov. Trans.*, vol. iv. p. 221, *et seq.*) Dr. Wise thus describes this injurious custom:

"Each miner having taken his portion of the blunted tools on his back, amounting in weight perhaps to from fifteen to twenty pounds, the most active generally takes the lead, and at whatever pace, is closely followed, along the horizontal galleries and up the perpendicular ascent of the ladders, by the rest of the party, not only from their desire to keep together, but to avoid a certain slur which would attach to any one falling in the rear. The rapid pace at which they usually set off soon hurries the circulation and respiration, and frequently to such a degree as to give rise to the most distressing feelings. At this time each would slacken his course, although none dare propose to do so; and thus they go on till they reach "grass," when they find themselves completely overpowered by the excessive actions of the heart and lungs, so as to be wholly unable for some time to speak." (p. 223.)

*Diseases of the Lungs.* The diseases of the organs of respiration require a more extended consideration, embracing, as they do, some of the most important deviations from health which can engage the attention of the profession. The severity and danger of the acute diseases of these organs, the intractable nature of many of the more chronic affections, the entire failure of the best directed efforts at cure in the vast majority of the most important of the whole, tubercular phthisis, are serious calls upon the humanity of the physician to devote his judgment, his skill, his most unwearied attention, his most unremitting industry, to the investigation of every point which is in any way calculated to throw light upon this class of diseases. If the position taken by Sir James Clark, in his admirable treatise on Phthisis Pulmonalis, be granted,—and we believe that few if any will be found to contest it,—that it is the prevention of the disease to which our efforts must chiefly tend, if we would obtain any measure of success against this insidious destroyer, it is equally true that the developing of the external causes which exert an influence in its production must be an object of the highest practical importance. We feel convinced that much light will be thrown upon these causes, and their mode of operation in inducing the deposition of tubercles, not only in the pulmonary tissues, but in other textures and organs of the body, by the study of the varying conditions of the different localities in which they are found to prevail, as compared with those, if such there be, which enjoy a comparative immunity from them. In this point of view, statistical and topographical details are of the highest utility: on the present occasion, we regret that the documents before us, although highly valuable, are much less comprehensive than we could desire.

The total number of diseases of the lungs in the two localities of Worcester and the Land's End is 3086; the total number of cases of disease in the two districts, 18,082, giving 1 in 5.8 as the general ratio of prevalence. In the former district there are 1616 cases of diseased lungs returned, affording a ratio of 1 in 5.7; in the latter district there are 1470, affording a ratio of 1 in 6. The ratio of prevalence being so nearly the same in each district as the general ratio of the two together, any disproportion in the ratios of prevalence of particular forms of pulmonic disease becomes the more striking, as showing a positive tendency existing in one or other of the localities, independent of many sources of error, which under other circumstances tend to complicate and obscure conclusions of this kind. The following table shows the ratio of prevalence of phthisis, as compared with diseases of the lungs, and with diseases in general in these districts, and in some others:

| | Cases of Phthisis. | Cases of Diseases of Lungs. | Ratio of Phthisis. | Total Cases of Disease. | Ratio of Phthisis. |
|---|---|---|---|---|---|
| Land's End and Worcester, | 713 | 3086 | 1 in 4.3 | 18082 | 1 in 25.3 |
| Worcester . . . . | 416 | 1616 | 1... 3.9 | 9255 | 1... 22.2 |
| Land's End . . . . | 297 | 1470 | 1... 4.9 | 8827 | 1 .. 28.7 |
| Plymouth . . . . | 176 | ... | ... | 5648 | 1... 32.0 |
| Birmingham . . | 295 | ... | ... | 18663 | 1... 63.9 |
| Hobart Town . . | 202 | 2502 | 1... 12.3 | 30008 | 1... 148.5 |

From the foregoing tabular view, it appears that tubercular consumption including hæmoptysis, (which is in nearly every instance either a symptom merely of phthisis, or at least accompanied with and resulting from the presence of tubercles in the pulmonary organs,) is more prevalent in that part of the Vale of Severn of which Worcester is the centre than in the Land's End, or indeed any other of the localities included in the table. This is a remarkable fact; as, from what has been before observed with respect to scrofula, to which tubercular consumption must be considered as nearly allied, it appears that the causes of tubercular depositions, and of that variety of constitution in which they are found to occur, are perhaps to the full as prevalent in the one locality as in the other. The greater prevalence of the external forms of scrofula in the Land's End district would, indeed, probably more than counterbalance the difference existing between the two localities in regard to the internal forms of the disease, and more especially phthisis. This, however, is not the case with the town of Birmingham, in which the ratio of prevalence of phthisis, as compared with other diseases, is as low as 1 in 63.9; for the proportion of external scrofula is not more than 1 in 162; a much lower ratio than either at Worcester, where it is 1 in 90, or in the Land's End, where it is 1 in 35; so that, in this large manufacturing town, notwithstanding the close crowding of the population and the nature of their avocations, there would appear to be a much less degree of prevalence of this fatal disease than in almost any other part of the kingdom. This is a statistical fact of vast import, if a more extended survey of the conditions of disease should confirm its accuracy; and we would here call the attention of the profession in Birmingham to the subject, with the view of inducing some of its members to undertake the labour of drawing up a full topographical account of that town and its immediate neighbourhood, with an especial reference to the degree of prevalence of the different forms of tubercular disease. It is very possible that some peculiar causes may have unduly modified the results of Mr. Parsons: at least, we find a very different result in a report by Dr. Ward in a subsequent volume of the Transactions, (*Prov. Trans.*, vol. v. p. 407,) viz. only 1 in 10. It is proper to observe, however, that the numbers included in Dr. W.'s report were very few in number (585) compared with those in the Tables of Mr. Parsons.* The proportion of phthisis in London is even greater than at Worcester, amounting to 1 in 19.7. In Plymouth also, according to Dr. Woolcombe, it is as high as 1 in 19.6: this ratio, however, it will be observed, is at variance with that obtained from Dr. Blackmore's Tables, which give the proportion of 1 in 32. The ratio of prevalence of scrofula in the same town, according to Dr. Blackmore, is 1 in 73. In Bolton le Moors, according to Dr. Black's Tables, (*Edin. Med. and Surg. Journ.*, vol. xxv. p. 444,) it is 1 in 21.9; being somewhat higher than at Worcester, though not equalling the rate of prevalence in London.

A due consideration of the preceding details will, we think, induce the physician to pause before he recommends the town of Penzance, and possibly other parts of the southern coast, as a residence for phthisical patients; for, however the equable nature of the climate may in some respects be suited to the consumptive invalid, the propriety of placing those who are either already affected with, or in any way predisposed to, tubercular phthisis in a locality where the disease is itself so prevalent, and the tendency to it so strongly marked, must be very questionable. Van Diemen's Land would seem to be a much more eligible situation to which those who are of a consumptive tendency might be sent; for the comparatively small number of cases of the disease, (only 202 out of upwards of 30,000,) and the low ratio of prevalence, not only with respect to diseases in general but, what is perhaps in this case a much more severe criterion, with regard to diseases of the pulmonary organs, is a fact worthy of attention, and one which speaks very favorably for the climate of this rising colony.

Sir James Clark estimates the relative mortality from this disease, both in

* Mr. Parsons' early death is a loss, not only to Birmingham but to the profession of which he was so valuable and esteemed a member. He was equally distinguished for his talents and moral worth.

France and England, at 1 in 3; Dr. Forbes estimates it, for the Land's End alone, at 1 in 4.3. At Carlisle, it is 1 in 7.5; at Plymouth, according to Dr. Woolcombe, 1 in 4.1, according to Dr. Blackmore, 1 in 6; giving as a mean for this locality 1 in 5. At London, it is 1 in 4.2; at Birmingham, according to Mr. Parsons, as deduced from observations for the years 1833, 34, and 35, 1 in 5.3. At Malvern, according to Mr. Addison, of 33 deaths, 7, or 1 in 4.7, were from phthisis. This gives a general average of 1 in 5.16, instead of 1 in 3, so that Sir J. Clark's estimate, at least as respects England, is probably too high. It is perhaps worthy of notice that, of these six localities, the ratio of mortality from phthisis is less than the mean at Carlisle and Birmingham, that is, in the two which are situated more to the north, and which may therefore be presumed to have a colder and more rigorous climate; while in the warmer climates of London, Plymouth, and the Land's End, it is greater than the mean. How far extraneous circumstances may have operated in the introduction of error into these conclusions we are not in a condition to form an opinion; but, of 155 deaths which occurred at the Worcester Dispensary, during the years 1828 to 1831 inclusive, 62, or 1 in 2.5, were from phthisis, (*Midland Medical Rpeorter,*) which proportion, if taken into the general account, would give a mean ratio of 1 in 4.75, instead of 1 in 5.16. This, however, is obviously an erroneous estimate of the relative mortality from phthisis, as compared with other diseases for Worcester, since it is evident, from a comparison of the columns 2 and 3 of the table given at page 8, that some regulation as to the admission of these cases exists at the Infirmary of that city, and that, therefore, a greater number than the just proportion are admitted at the Dispensary: the relative mortality from this cause at this last institution is therefore probably higher than the true ratio for the city and its immediate neighbourhood. The number of cases denoted *phthisis* admitted at the Worcester Dispensary during this period, (from 1828 to 1831 inclusive,) was 235; the number of deaths from this cause 62, or 1 in 3.8: the proportion of deaths to the cases at Birmingham during the years 1833, 34, and 35, was, according to Mr. Parsons, 1 in 1.38; a difference which leads to the supposition that either many cases of tubercular phthisis in its earlier stages are classed under other affections at the Birmingham Infirmary, (a supposition which is supported by the number of cases of hæmoptoe which occur in the tabular returns of that Institution), or that some of those which have been so classed in the tables of the Worcester Dispensary were not the true tubercular consumption, but some other form of chronic pulmonary disease.

Bronchitis, catarrh, and other affections of the pulmonary mucous membrane form a large proportion of the diseases to which the inhabitants, both of the Land's End and of the city of Worcester, are liable. This is probably to be attributed, in part at least, to the humidity and relaxing nature of the climate; but, however a predisposition to these diseases may be thus acquired, the inhalation of particles of dust, forming a continuous source of irritation to the delicate membrane lining the bronchial tubes and cells, must, in either of these situations, be considered as a chief exciting cause in the production of this class of affections. The glovers of Worcester, no less than the miners of Cornwall, are constantly breathing an atmosphere loaded with the fine particles resulting from the mechanical disintegration which takes place in many branches of their respective employments, and the effects of such a practice are too evident to require here to be pointed out. It may not be uninteresting to compare the proportional prevalence of bronchial disease with that of tubercular phthisis, and we therefore subjoin a tabular arrangement, similar to that which we have already given of this latter disease.

| | Cases of Bronchitis, &c. | Cases of Diseased Lungs. | Ratio of Bronchitis. | Cases of General Disease. | Ratio of Bronchitis. |
|---|---|---|---|---|---|
| Land's End and Worcester, | 1337 | 3086 | 1 in 2.3 | 18082 | 1 in 13.5 |
| Worcester . . . | 736 | 1616 | 1 ... 2.2 | 9255 | 1 ... 12.5 |
| Land's End . . . | 601 | 1470 | 1 ... 2.4 | 8827 | 1 .. 14.6 |
| Plymouth . . . | 319 | ... | ... | 5648 | 1 ... 14.5 |
| Birmingham . . | 3541 | .. | ... | 18663 | 1 ... 5.3 |
| Ditto, excluding 684 cases of influenza | 2857 | ... | ... | 18663 | 1 ... 6.5 |
| Hobart Town . . | 1784 | 2502 | 1 ...1.4 | 30008 | 1 ... 16.8 |

A very curious, and (if at all approaching to correctness) a very important feature in the results exhibited in the preceding table, is the close approximation of the ratios of prevalence in four out of five of these localities. The mean ratio of prevalence of these four is 1 in 14.6, or precisely that of the Land's End. Now, the only circumstances which, as far as we know, are common to three of these localities,—namely, the Land's End, Plymouth, and Worcester,—appear to be a tolerably equable temperature, and an atmosphere frequently charged with humidity; and which, therefore, however they may seem to dispose to catarrhal diseases, as appears from the great prevalence of this class of affections in such situations, may yet probably exert a counteracting effect upon other causes presumed to be influential in inducing disease of the mucous membranes; as, for instance, upon the irritation arising from the presence of particles of dust in the respired atmosphere. That such may be the case seems to be rendered still more probable by the extremely high ratio of prevalence of bronchial diseases in the town of Birmingham, where they constitute 1 in 5.3, or, omitting the cases of influenza, 1 in 6.5, of the whole number of diseases. No doubt this high proportion is owing, in part, to the still more irritating nature of the particles of dust with which the air in many of the manufactories is charged, consisting of fine metallic particles, exerting both a chemical and mechanical effect upon the secretions and organization of the delicate membrane with which they come in contact; whereas, in Worcester, the dust consists chiefly of the finer particles of leather and minute angular fragments of the pumice-stone used in the grounding of the skins; and, in the Land's End, of particles of earth and ore, resulting from the operations of blasting, picking, &c. carried on in the mines. This cause, however, independent of any variation in the mechanical or chemical nature of the particles of dust, must be materially lessened by a humid state of the atmosphere, and may therefore be expected to exercise a more deleterious influence in the dry, heated air of the manufactories of a situation exposed to no extraneous sources of moisture, like Birmingham, than in one in which the air is preserved in a state of almost constant humidity, as occurs in the mines of Cornwall. We must still, however, bear in mind that both the Cornwall and Worcester ratios are calculated from a series of cases which embrace a considerable proportion of the agricultural population; while the ratio deduced from the Birmingham Reports refers to a population almost exclusively manufacturing. One other source to which the difference may possibly in part be owing requires to be noticed, and that is, the probability that many of the cases arranged as chronic bronchitis and catarrh in the Birmingham Tables may have been cases of tubercular phthisis; a supposition to which the extremely low ratio of prevalence of this latter disease, as deduced from the same tables, gives considerable support.

The length to which our observations have extended precludes us from extracting any detailed account of the differences which may exist in the nature of the phenomena presented by these affections as they occur in the several localities. The cases at the conclusion of the paper on the Land's End, in the fourth volume of the Transactions, may however be referred to for an illustration of the principal varieties of the *Miner's consumption*, as the disease is there emphatically called.

*Diseases of the Abdomen.* Many of these afford room for observation; but we can only remark at present that the difference in the Land's End and Vale of Severn, in respect to the cases of dyspepsia and hepatitis, which nearly compensate each other, are probably to be explained by the different views which different practitioners take of this class of diseases; some including under the term dyspepsia cases to which others, from a tender state of the epigastric region, combined with evidences of derangement in the hepatic functions, would probably give the name of chronic hepatitis.

The large proportion of cases marked diarrhœa, cholera, and dysentery, in the Worcester Tables, arises from the cases of the epidemic cholera being included, and not from any real excess of disease of that description. Taking the ratio of prevalence afforded by the Dispensary Tables from 1828 to 1831, which was previous to the appearance of the epidemic, we have the proportion of 1 in 31.4; and, making allowance for the cases of malignant cholera and epidemic diarrhœa included in the Penzance Tables, which the subsequent remarks of Dr. Forbes (*Prov. Trans.*, vol. iv. pp. 180, 181,) enable us to do, we obtain a proportion very nearly the same.

*Diseases of the Urinary Organs.* There is a much larger proportion of these diseases in the Penzance Tables than in those of the Vale of Severn; the ratio of prevalence in the former case being 1 in 83; in the latter, at Worcester and its vicinity, only 1 in 185. In Bristol, of 700 medical cases, there were only 6 of disease of the urinary organs, or 1 in 116.6; and, in Malvern, only 2 out of 578, or 1 in 289. The cause of this does not appear, but the cases are too few to derive any positive inferences from.

*Uterine Diseases.* Here, again, there is a ratio of prevalence in the Land's End district which differs greatly from that of Worcester and the neighbouring country; the respective proportions being in the Land's End 1 in 14.1, in Worcester only 1 in 39. At Bristol the proportion, as deduced from the 700 cases before referred to, is 1 in 26.9, and at Malvern as high as 1 in 13.7. Both these latter proportions are, however, obtained from selected cases; the ratio of Bristol, as has been already remarked, including only the medical cases, and the ratio of Malvern omitting the slighter cases, as well as those more strictly surgical. Dr. Forbes makes the following observations with respect to this subject:

"The only diseases of this class in the Dispensary Tables that require notice are those comprehended under the head of chlorosis and amenorrhœa. It will be observed that the proportion of cases so denominated is extremely great, being, in fact, nearly three times as great as in London, and more than one-half more than in Plymouth, It will also be observed, that the disproportion is entirely owing to the very great number of cases thus distributed in the first column of the table. Taking the two last columns only, we find that the proportion of such cases (even when those classed under dysmenorrhœa are included) to the total diseases, during the same period, instead of being 1 in 21, is only 1 in 25, or, excluding dysmenorrhœa, 1 in 30. Still even this is a very large proportion, and I can only account for it by supposing that every case presenting any deficiency in the catamenial discharge was arranged under this head, without considering how frequently the amenorrhœa is a mere symptom of other diseases. I am borne out in this supposition by the result furnished by the second column, which gives the proportion only as 1 in 52. I do not doubt but that, if as rigid analyses had been instituted during the other years as was done by myself in classifying the cases during these three years, the general result would have approached nearer to that last stated. It is, however, not improbable that these affections are more prevalent in this district, although not to the extent alleged; and that this increased prevalence may be owing to the greater amount of dyspeptic affections, of the more inveterate forms of which amenorrhœa is often a consequence." (*Prov. Trans.*, vol. iv. p. 212.)

*Chronic Eruptions.* The chronic eruptive diseases constitute another series of morbid affections, which are proportionally more prevalent in the Land's End. These are attributed—and justly so, we have no doubt, —to defective nutriment and clothing, and general want of cleanliness, on the part of the inhabitants. The ratio of prevalence is 1 in 16.9; whereas, in Worcester and its vicinity, it does not amount to more than 1 in 40. The proportion in Van Diemen's Land is almost exactly double, being 1 in 20.5.

*Bronchocele.* This is another disease to which we must briefly allude, as it is one which has been supposed to be particularly prevalent in the midland counties. At Worcester, the number of cases reported is 47 out of 9255, giving the proportion of 1 in 198; in the Penzance Tables we have only seven cases, or 1 in 1261. The ratio of prevalence, 1 in 198, must be very considerably below the true ratio, as, from the trifling inconvenience experienced from this affection in its earlier stages and slighter forms, it is probably only the more urgent cases,—those in which either great deformity or some derangement of health results,—which become the subjects of treatment at public institutions.

We must not bring these observations to a close without directing attention to the excellent Statistical Observations on the Medical Charities of England and Ireland, by Dr. Walker, of Huddersfield, which deserve a fuller and more attentive consideration than we could possibly devote to them. For similar reasons we are obliged to pass over some other papers which require notice, but we cannot omit to refer to Dr. Black's paper on the Medical Topography of Bolton le Moors and its Neighbourhood, in the last volume of the Transactions, as possessing peculiar claims upon the attention of our readers. It is to be regretted, however, that the author of this excellent statistical sketch has not entered upon the more strictly medical part of it to an extent commensurate with its other portions, and especially that he has omitted to subjoin tables of disease. Should he have it in his power to rectify these omissions upon some future occasion, he will materially add to the value of what he has already written.

In conclusion we must observe that, although we could point to some papers in these volumes hardly suited to the object of the Association, or worthy of its reputation, we regard the majority of them as calculated at once to do honour to and to elevate the character of the provincial practitioner: they are not of the mere passing interest which is likely to excite the attention of the superficial reader, but require a deeper insight into the philosophy of medicine than either suits the purposes or is agreeable to the taste of the literary charlatan. To such an individual we can readily believe that the study required to develop the true principles of science must prove a wearisome and distasteful task: he cannot appreciate, because he is, both morally and intellectually, incapable of comprehending the solid truths upon which alone the superstructure of real, substantial knowledge can be erected.

---

